# Effectiveness, Acceptability, and Feasibility of a Telehealth HIV Pre-Exposure Prophylaxis Care Intervention Among Young Cisgender Men and Transgender Women Who Have Sex With Men: Protocol for a Randomized Controlled Trial

**DOI:** 10.2196/47932

**Published:** 2023-09-15

**Authors:** Rebecca A Braun, Rebecca K Erenrich, Karin K Coyle, Thuan-Huong P Doan, Jeffrey D Klausner

**Affiliations:** 1 ETR Associates Scotts Valley, CA United States; 2 University of California, Los Angeles Los Angeles, CA United States; 3 Keck School of Medicine, University of Southern California Los Angeles, CA United States

**Keywords:** youth, young people, men who have sex with men, transgender women, HIV, pre-exposure prophylaxis, PrEP, effectiveness, acceptability, feasibility, telehealth, web-based intervention, digital intervention, randomized controlled trial, RCT

## Abstract

**Background:**

Despite its promise for HIV prevention, the uptake of pre-exposure prophylaxis (PrEP) has been slow, and there have been substantial inequities in PrEP access. Young men who have sex with men and transgender women of color are most in need of PrEP and least likely to have that need fulfilled. PrEP telehealth care, which provides remote PrEP care via electronic communication, seems well suited to address several of the challenges of PrEP provision, including discomfort with stigmatizing and difficult-to-access health care systems, transportation challenges, and privacy concerns, and address disparities in PrEP access. Research suggests that PrEP telehealth care has promise and is a favored option for many prospective recipients of PrEP. However, despite growing attention to telehealth approaches as an avenue for increasing access to PrEP amidst the COVID-19 pandemic, there have been no published randomized controlled trials (RCTs) on PrEP telehealth care to date, making it difficult to draw strong conclusions about the advantages or disadvantages of telehealth compared with usual PrEP care. We developed PrEPTECH, a telehealth intervention that focuses specifically on alleviating issues of stigma, access, cost, and confidentiality for young people with risk factors for HIV infection who are seeking PrEP care. Leveraging data from the 2017 observational pilot study, we redesigned and enhanced PrEPTECH.

**Objective:**

This study aims to assess the effectiveness, acceptability, and feasibility of a telehealth HIV PrEP care intervention, PrEPTECH, in increasing PrEP uptake.

**Methods:**

This is the protocol for an RCT of young cisgender men and transgender women who have sex with men in 4 regions within the United States: the San Francisco Bay Area, California; Los Angeles County, California; Miami-Dade County, Florida; and Broward County, Florida. Participants in the intervention arm received access to a web-based telehealth program, PrEPTECH, which offers a fully web-based pathway to PrEP, whereas those in the control arm received access to a dynamic web page containing publicly available informational resources about PrEP. Follow-up data collection occurred at 3 and 6 months. An analysis will be conducted on outcomes, including PrEP initiation, persistence, adherence, coverage, and medication prescription, as well as PrEPTECH acceptability and feasibility.

**Results:**

The study was funded in 2019 and received institutional review board approval in 2020. The PrEPTECH intervention was developed over the next 1.5 years. Study recruitment was launched in February 2022 and completed in September 2022, with 229 participants recruited in total. Data collection was completed in April 2023.

**Conclusions:**

The results of this RCT will offer valuable evidence regarding the effectiveness, acceptability, and feasibility of telehealth HIV PrEP care interventions among young cisgender men and transgender women who have sex with men.

**Trial Registration:**

ClinicalTrials.gov NCT04902820; https://clinicaltrials.gov/ct2/show/NCT04902820

**International Registered Report Identifier (IRRID):**

PRR1-10.2196/47932

## Introduction

### Background

The use of antiretroviral medications as pre-exposure prophylaxis (PrEP) is an effective biomedical HIV prevention approach first approved by the US Food and Drug Administration in 2012 [1]. Copious research has established that HIV PrEP presents few significant safety risks and substantially reduces the likelihood of contracting an HIV infection in individuals at risk when taken consistently. Research has confirmed the effectiveness of PrEP for adolescent men who have sex with men [2] and transgender women [3] when it is taken consistently.

Despite its promise for HIV prevention [4,5], the uptake of PrEP has been slow, although it has improved recently, and there have been substantial inequities in PrEP access. PrEP is indicated for an estimated 1.2 million people in the United States according to the Centers for Disease Control and Prevention, but in 2020, only 24.7% of those for whom PrEP was recommended were prescribed it (compared with approximately 3% in 2015) [6]. There are substantial racial and gender disparities in PrEP uptake. Specifically, 4 in 10 Black/African American men who have sex with men and 2 in 10 Hispanic/Latino men who have sex with men will be diagnosed with HIV infection in their lifetime, compared with only 1 in 10 White men who have sex with men [7]. However, PrEP coverage (the percentage of those with an indication for PrEP who received a PrEP prescription) ranges from 65.4% among White people to 15.7% among Hispanic/Latino people to 9.1% among Black/African American people [6]. The prevalence of HIV among transgender women in the United States is 14%, over 30 times higher than that in the US population overall [8], but a nationally representative study of transgender people found that 3% of transgender respondents who were having sex with cisgender men or transgender women were taking PrEP [9]. People aged 16 to 24 years have the lowest rates of PrEP uptake of any age group, with only 15.6% of those with an indication for PrEP receiving a prescription for PrEP in 2020 (Centers for Disease Control and Prevention) [6]. Adherence to and persistence in the use of PrEP are also less favorable among Black/African American men who have sex with men and youth [10,11]. There is a dearth of research on the rates of adherence to and continuation of PrEP among transgender women [12]. In sum, young people of color, particularly young men who have sex with men and transgender women of color, are most in need of PrEP access and least likely to have that need fulfilled.

Researchers have identified a range of factors that may interfere with initiating and adhering to PrEP, including a lack of awareness or knowledge of PrEP, poverty, health system inaccessibility, stigma, concerns about side effects, competing stressors, and low HIV risk perception [13-17]. Health system factors that impede PrEP access include primary care providers’ lack of familiarity or comfort with dispensing PrEP care, racial and ethnic bias, homophobia, and transphobia among health care providers, and distrust in the medical system among potential PrEP recipients [18]. Logistical difficulties, such as the lack of transportation, prevent people seeking PrEP from reaching PrEP clinics, which are sparser in areas with higher poverty, larger Black/African American and Hispanic/Latino populations, and lower rates of insurance coverage [19]. Concerns about insurance and the ability to pay can also present obstacles. Obstacles to the continuation of PrEP include health system barriers, acute medication side effects, decreased sexual risk-taking and perceived HIV risk, and health system factors such as cost and difficulty attending appointments [20-22].

Telehealth or telemedicine, the use of electronic communication to improve patient health [23], seems well suited to addressing many of the challenges of PrEP dissemination and has become increasingly relevant during the COVID-19 pandemic [24]. Telehealth interventions may remove obstacles within the health care system that complicate beginning and remaining on PrEP. Telehealth may simplify the process of obtaining PrEP and lessen the impact of stigma by allowing participants to access PrEP more privately [25]. A survey of a nationwide cohort of men who have sex with men who were HIV negative and had not initiated PrEP found that the vast majority said that they would prefer to undergo follow-up home testing and receive mail-delivered PrEP after an initial in-person appointment to start PrEP care (termed “home-based care” in the study) over regular follow-up at a clinic [26]. Men who were younger or reported being distant from the nearest lesbian, gay, bisexual, transgender, and queer (LGBTQ+)–friendly health care provider were more likely to prefer home-based PrEP care, as were those who had more potential exposure to HIV infection and a stronger intention to start PrEP. A meta-ethnography reviewing data from 11 unique qualitative studies of men who have sex with men in the United States concluded that structural interventions, including telehealth for PrEP, may help circumvent barriers to PrEP for some men who have sex with men, such as difficulty obtaining transportation and maintaining engagement with treatment [14].

Many PrEP care providers, such as health care providers, have generally turned to telehealth approaches because of the evident need for alternatives to in-person care since the beginning of the COVID-19 pandemic [27,28]. A small study of patient satisfaction following a clinic’s transition to telehealth-delivered PrEP care midpandemic found a preference for telehealth PrEP visits over traditional in-clinic PrEP visits [29]. The ranks of commercial and nonprofit providers of PrEP telehealth care have grown in the past few years, with multiple nationwide companies providing telehealth care, alongside numerous local efforts, in the United States [22].

### PrEP Telehealth Delivery

Despite growing attention to telehealth approaches as an avenue for dispensing PrEP, research on PrEP telehealth delivery has been mostly limited to general reports on implementation [30]. PrEP telehealth projects that researchers have reported on include Iowa TelePrEP, a public health–partnered regional PrEP telehealth project; a telehealth extension of a Seattle PrEP clinic; a San Francisco clinic transitioning to a telehealth-focused model in the midst of the COVID-19 pandemic; and PlushCare and Nurx, 2 commercial providers of telehealth PrEP care. Research on user experience and preferences has largely found that patients are receptive to PrEP telehealth and largely report positive experiences. A qualitative report on user experience with the Nurx telehealth PrEP service found strongly favorable responses and appreciation for the service’s achievement of a balance between efficiency (simplicity, speed, and convenience) and humanity (feeling of personalized service, connection, and care) [31]. Research on the San Francisco clinic transitioning to telehealth found that telehealth-based care was preferred by many prospective PrEP users because of the convenience and perceived safety of telehealth, whereas technical barriers and unfamiliarity of the platform were considered disadvantages of PrEP telehealth [32]. In addition, in a PrEP telehealth pilot that enrolled 40 participants and provided PrEP to 20 participants, only 13% (n=5) of the participants said that they would have preferred in-person–only PrEP care to exclusive telehealth care or a blend of telehealth and in-person care [33].

The limited research on the rates of PrEP uptake in the PrEP telehealth setting suggests that PrEP telehealth can be effective. Most Iowa TelePrEP clients who completed video-based telehealth visits (91%) started PrEP, and the intervention had a 61% retention rate at 6 months, indicating that widely available technology can be a viable tool for delivering PrEP in small urban and rural settings [25]. In the mixed methods evaluation of their laboratory testing site versus home testing options for routine monitoring, home kit use was associated with higher completion of some tests (extragenital swabs) and lower completion of others (blood tests); self-efficacy in using kits, time, convenience, privacy, and confidentiality were the primary factors influencing the use of home test kits [26]. In addition, a report on the telehealth extension of a Seattle PrEP clinic, which described implementation and compared 10 patients who opted to receive telehealth care with 38 patients who did not opt to receive telehealth care, found no significant differences between the groups in the proportion of participants prescribed PrEP, attendance at their first follow-up visit, and adherence at 1 month but found low rates of attendance at the 3-month visit among patients who opted to receive telehealth PrEP who had not yet been referred to another health care provider for PrEP.

We developed PrEPTECH, a telehealth intervention that focuses specifically on alleviating issues of stigma, access, cost, adherence, and confidentiality in young people with risk factors for HIV infection who are seeking PrEP care. A 2017 observational pilot study of PrEPTECH found that the intervention helped users bypass some of the documented barriers to PrEP access, uptake, and adherence [34]. The 25 men who have sex with men who were HIV negative, were aged 18 to 25 years, were from the San Francisco Bay Area, and enrolled in the pilot study reported high satisfaction with PrEPTECH, and over 85% expressed preference for telehealth-delivered PrEP care over clinic-delivered PrEP care. Leveraging the data from the pilot study, we redesigned and enhanced PrEPTECH and are now testing whether the updated platform enhances PrEP uptake among young cisgender men and transgender women who have sex with men through a randomized controlled trial (RCT). Ultimately, this study will add to the growing evidence base about the effectiveness, feasibility, and acceptability of telehealth HIV PrEP care.

## Methods

### Study Design

This study is an RCT for assessing the effectiveness, acceptability, and feasibility of a telehealth HIV PrEP care intervention, PrEPTECH, in increasing PrEP uptake over 6 months among young cisgender men and transgender women who have sex with men in 4 regions within the United States, namely the San Francisco Bay Area, California; Los Angeles County, California; Miami-Dade County, Florida; and Broward County, Florida, when compared with those of standardized web-based PrEP information resources.

### Study Objectives

The study has two primary objectives: (1) to test the effectiveness of a telehealth intervention, PrEPTECH, in enhancing PrEP uptake in comparison with that of a condition in which only standardized web-based PrEP information resources are provided and (2) to assess the acceptability and feasibility of PrEPTECH as a telehealth HIV PrEP care intervention for young cisgender men and transgender women who have sex with men.

In addition, the study has two secondary objectives: (1) to test the effectiveness of a telehealth intervention, PrEPTECH, in improving adherence to PrEP in comparison with that of a condition in which only web-based standardized PrEP information resources are provided and (2) to examine the moderators of PrEP use, including HIV risk perception, PrEP attitudes, sexual behaviors associated with HIV risk, and insurance status.

### Study Hypotheses

We hypothesize that participants in the intervention arm will show higher rates of PrEP initiation at both 3 and 6 months than those in the control arm. We also hypothesize that PrEPTECH is an acceptable and feasible telehealth HIV PrEP care intervention for young cisgender men and transgender women who have sex with men.

### Study Setting

The study will be completed digitally among self-referred participants living in the San Francisco Bay Area, California (the 9 counties of Alameda, Contra Costa, Marin, Napa, San Mateo, Santa Clara, Solano, Sonoma, and San Francisco); Los Angeles County, California; Miami-Dade County, Florida; or Broward County, Florida.

### Sample Size

We calculated the sample size to provide adequate statistical power for our first objective, which is to test the effectiveness of a telehealth intervention, PrEPTECH, in enhancing PrEP uptake. Data suggest that PrEP uptake among relevant populations is low. Only 16% of young people aged 16 to 24 years use PrEP [6]. We also reviewed recent RCTs aimed at increasing PrEP uptake in comparable study populations. A pilot study of linkage to PrEP care among men who have sex with men and transgender women identified as partners of people diagnosed with a sexually transmitted infection (STI) found an uptake rate of PrEP of 11% among controls receiving standard HIV prevention programming, including PrEP care [31]. In a pilot trial of a 2-session motivational interviewing–based behavioral intervention, 28% of controls receiving treatment as usual at an urban STI clinic received a PrEP prescription [35]. Furthermore, 2 RCTs of web-based interventions for increasing PrEP uptake among sexual minorities with published protocols estimated anticipated control arm uptake ranging from 2% to 15% for the purpose of their power analyses [36,37].

Using this recent research on PrEP uptake in relevant populations and comparable research projects, including ongoing RCTs of digital interventions, we assumed 20% PrEP uptake in the control population. We proposed a minimum sample size target of 192 participants. Retaining 83.3% (160/192) of these participants at the 90-day and 180-day follow-up was our benchmark, as a loss to follow-up of greater than 20% in RCTs is seen as potentially introducing a source of bias [38]. We calculated statistical power using the following assumptions: power (1 – β)=.80, significance level (α)=.05, PrEP uptake in the control group of 20%, and PrEP uptake in the intervention group of ≥40%. The power analysis results indicate a required sample size target of 96 per group or 80 per group after anticipated attrition.

### Eligibility Criteria

To be eligible, participants must live in the San Francisco Bay Area, California (the 9 counties of Alameda, Contra Costa, Marin, Napa, San Mateo, Santa Clara, Solano, Sonoma, and San Francisco); Los Angeles County, California; Miami-Dade County, Florida; or Broward County, Florida. Participants must self-report having sex with men and identify as a cisgender male and be aged 15 to 17 years (in California only; minors in Broward or Miami-Dade County will not be eligible), identify as a cisgender male and be aged 18 to 27 years, or identify as a transgender female (or assigned male at birth and identify as a woman) and be aged 18 to 27 years (transgender females outside this age range will not be not eligible). In addition, participants, in keeping with guidelines for the provision of PrEP for sexual risk factors, must qualify for PrEP by self-reporting being diagnosed with an STI (including chlamydia, genital herpes, gonorrhea, syphilis, genital warts, human papilloma virus infection, and trichomoniasis) in the past 6 months or self-reporting condomless anal intercourse in the past 90 days [39]. Having a current known HIV-positive sexual partner and an indication for PrEP based on injection drug use were not included as eligibility criteria because of their expected rarity in our young study population. Among individuals aged 21 to 30 years, 1.5% reported ever injecting a drug not under a physician’s orders and only 0.5% reported sharing needles in this way in their lifetime [40], and in a study of youths aged 12 to 24 years at high risk for HIV infection, only 7.7% reported ever having sex with a person living with HIV [41]. Only participants who attested to feeling comfortable reading and speaking in English were included, as, owing to development costs, creating a version of the website in Spanish was not feasible and other services associated with the intervention, including laboratory and pharmacy services, were in the English language only. Participants are not eligible if they have taken PrEP in the past 30 days; have ever been diagnosed with HIV infection, hepatitis B infection, chronic kidney, liver, or bone disease; or do not meet the eligibility criteria.

### Recruitment and Randomization

To recruit potential participants, we contracted with an LGBTQ+ marketing and communications company to run paid advertising campaigns on social media platforms (eg, Facebook [Meta Platforms, Inc] and Instagram [Meta Platforms, Inc]) and LGBTQ+ dating apps (eg, Grindr [Kunlun Tech Co Ltd], Scruff [Perry Street Software Inc], Jack’d [Perry Street Software Inc], and Adam4Adam [A4A Network Inc]). The advertisements were targeted to the age and geographic eligibility criteria of the study and included a diverse array of men who have sex with men and transgender people, prominently featuring Black/African American and Hispanic/Latino individuals.

In addition to web-based recruitment, we identified community-based organization partners in each geographic location of the study and paid them to conduct web-based and in-person outreach. Community partners received printed assets (eg, palm cards and flyers) with the link to the study website and a QR code linked to the study website, as well as social media assets, including graphics sized for multiple social media platforms (eg, Instagram, Facebook, and Twitter [Twitter, Inc]), suggested captions, and potential hashtags.

Upon recruitment, prospective participants were directed to the study website and asked to complete an eligibility screener. Participants who were eligible completed an informed consent process.

To ensure that participants understood the risks that PrEP and participation in this study entail, the informed consent text outlined the risks in a detailed plain language summary. Taking advantage of the functionality available in a web-based setting, comprehension questions were incorporated into the informed consent text; prospective participants could not advance through the document until each question was correctly answered. We received a waiver of parental permission and, therefore, did not require or seek parental consent for minor participants. Participants who correctly answered the comprehension questions and consented to participate were brought to a baseline survey; upon completion, they were randomized into one of the two study arms. Only those who met the eligibility criteria and consented to participate were included in the study.

Eligible participants were randomized equally between the intervention arm, a web-based telehealth program offering a fully web-based pathway to PrEP, and the control arm, a dynamic web page containing publicly available informational resources about PrEP. We intended to ensure equal allocation of participants across the 4 study areas and equal numbers of adolescent cisgender males and adult transgender females in each study arm (approximately 20 individuals belonging to each subgroup in each study condition).

### Community Advisory Board

During the development of the PrEPTECH intervention, we convened a 6-member community advisory board (CAB), composed of individuals who met the age and geographic eligibility criteria for the study and identified as a cisgender male or transgender female. Over the course of 5 web-based meetings, CAB members provided critical input and feedback on visual design, user experience, survey tools, recruitment approaches, and marketing and communication efforts. CAB members provided general guidance on how to appeal to prospective participants, suggesting that we highlight the incentive, potential to help the community through participation in this research, and benefits of PrEP. They offered the tagline used in the study’s marketing materials, “PrEP on your own terms,” following their discussion of the value of emphasizing PrEP as a personal exercise of autonomy, and, over several sessions, provided feedback on and later selected images and designs proposed by an in-house graphic designer for use on the PrEPTECH website and marketing materials. Each CAB member also took a draft survey and submitted feedback on any questions that they felt confused by or uncomfortable with; we also solicited input from the CAB about important topics that might be missing from the survey and on potential barriers to PrEP access that should be asked about.

### Intervention

Participants allocated to the intervention arm of the study gained access to a web-based telehealth platform called PrEPTECH, which provides PrEP education via self-guided reading material, access to laboratory testing for PrEP eligibility delivered to a participant’s home, and optional SMS text messages or email reminders to aid in medication adherence. PrEPTECH also provides access to telehealth care and PrEP prescriptions for eligible patients asynchronously through a web-based form or synchronously through telephone appointments with the study clinician, an infectious disease physician with expertise in PrEP care licensed in California and Florida. The PrEPTECH intervention tested here is an extension of the piloted PrEPTECH intervention. A brief report on the results of the pilot also discusses the development of the initial intervention by a multidisciplinary team in concert with a previous CAB [42]. It was adjusted to serve a larger group and to be completely web-based. In the second version of PrEPTECH, we incorporated a home laboratory testing kit service, built a platform for offering web-based health evaluations, and developed back-end systems for PrEPTECH to enable the clinical team to review submitted information and communicate with participants. The site was also redesigned for mobile platform compatibility.

All participants who completed at-home laboratory testing and an asynchronous or telephone appointment with the study clinician for whom PrEP was indicated received a prescription for Truvada (emtricitabine, 200 mg or tenofovir disoproxil fumarate, 300 mg). Adult cisgender male participants aged 18 to 27 years received a free 30-day supply of PrEP and subsequently had to pay for PrEP medication through insurance, through patient assistance programs, or out of pocket, should they choose to continue taking PrEP. Adolescent cisgender male participants aged 15 to 17 years and adult transgender female participants aged 18 to 27 years received free PrEP medication for the duration of their 6-month participation in the study because these subgroups were not included in the pilot study of PrEPTECH. Knowing less about the suitability of this intervention for these susceptible populations, the study team opted to provide free medication to them for the duration of the study. PrEPTECH also provides support for signing up for PrEP access programs, prescription transfer, and coordination with community partners.

After randomization to the PrEPTECH group, participants entered the PrEP education section, which included information about use, side effects, and effectiveness, and completed quiz questions to ensure comprehension. Next, a myLAB Box home testing kit for HIV, hepatitis B, syphilis, creatinine, gonorrhea, and chlamydia was sent to an address of their choice (typically, their home) [43]. Participants collected the specimens required through swab and finger prick and sent them via a postage paid mailer. If the participant tested negative for HIV antibodies or another STI, the web-based results were made available to the participants, and they were prompted to complete a medical intake questionnaire. This questionnaire was reviewed by the study clinician to confirm that there were no contraindications to PrEP medication for the participant. Synchronous telehealth appointments (telephone or video calls) were provided to all adolescent participants because it was anticipated that they might have a greater need for explanation and guidance that could best be offered in this format. For all other participants, synchronous telehealth appointments were offered as needed or requested.

If the study clinician was satisfied that the participant was eligible, he would issue a 6-month prescription for PrEP. A free initial supply of PrEP would be filled by Ridgeway Pharmacy, a mail order pharmacy serving both California and Florida, and mailed to a location of the participant’s choice (typically, their home) [42]. For adult cisgender male participants, this initial shipment would be a 30-day supply of PrEP, but for adolescent cisgender male or adult transgender female participants, a 90-day free supply was sent. If a participant tested positive for HIV antibodies or another STI or any significant laboratory abnormalities were found, rather than reporting the results directly to the participant, a synchronous telehealth appointment would immediately be scheduled, and the study clinician would share the laboratory results, provide any necessary counseling, and refer the participant to a community provider for care. These participants would remain included in the study and would still be prompted to take the 90- and 180-day surveys. Then, 30 days after the prescription was sent, participants completed a second medical intake questionnaire, which was reviewed by the study clinician to assess any side effects or challenges encountered in taking PrEP. Participants could also request a synchronous appointment with the study clinician at any time during the study. Furthermore, 3 months (90 days) after enrollment, participants completed a second myLAB Box home testing kit for HIV, syphilis, gonorrhea, and chlamydia, as well as a third medical intake questionnaire, which was reviewed by the study clinician, with similar follow-up procedures as before. Follow-up laboratory tests did not include PrEP adherence measures such as hair sample or blood spot test, as they cannot be performed in the commercial laboratory with which we are working. Communication about steps toward accessing treatment is made through emails and texts, depending on the preferences expressed by the user, which can be indicated on the website itself.

Following their completion of the PrEP knowledge module, participants were queried about their insurance coverage and preferences with regard to paying for PrEP beyond their free supply. Using this information, the study staff worked with each participant to identify a means of payment, should the participant choose to continue taking PrEP. Participants could opt to use their existing insurance or pay out of pocket, in which case they would contact a pharmacy of their choice and request a transfer of the prescription. If they did not have insurance, they could seek coverage through Ready, Set, PrEP, a nationwide program that covers the cost of PrEP [44]. Ready, Set, PrEP allows care providers and patient navigators to sign up prospective participants. Participants would be given the option of signing up for Ready, Set, PrEP themselves, in which case the PrEPTECH platform provided detailed instructions for correctly completing their application, or they may authorize the study team staff to sign up on their behalf. Alternatively, if a participant opted for a community partner referral, they would be connected to an organization offering PrEP navigation in their geographic area. Near the end of a participant’s time in the study, additional information and offers of support for transitioning to community care were provided again, including referrals to community partners.

Sample images from the PrEPTECH intervention can be found in Multimedia Appendix 1.

### Control

Participants allocated to the control arm of the study gained access to a dynamic web page containing publicly available standardized informational resources about PrEP, including information on eligibility, use, side effects, effectiveness, access, and payment, as well as specific PrEP resources for transgender females. This web page also provides a list of alternative telehealth-based PrEP providers and contact information for community partners offering PrEP navigation in each geographic area.

The personal landing page for each participant was customized based on their study arm and subgroup (adolescent, adult transgender woman, or adult cisgender man who has sex with men). Participants allocated to the control condition could not access any of the PrEPTECH features, which were not displayed to them. A second panel on the control arm participants’ landing page shows what surveys they have taken and are currently eligible to take.

### Participant Timeline

**Figure 1:**
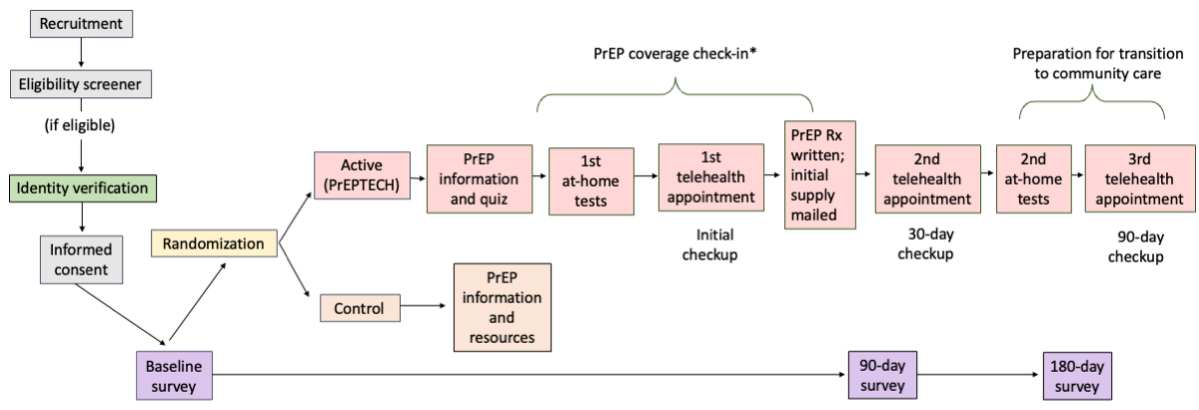
PrEPTECH study and intervention participant experience. *For adult men who have sex with men who are initially provided with a 30-day supply of free pre-exposure prophylaxis (PrEP), information and guidance on paying for PrEP (through insurance, patient access programs or self-pay) is provided concurrently with the provision of at-home laboratories and the initial telehealth appointment. Rx: prescription.

The entire 6-month web-based study is completed on the PrEPTECH website. A sequential illustration of participants’ experiences is shown in Figure 1.

1. Recruited prospective participants review web-based materials on the PrEPTECH website and elect whether to create a web account, which requires them to verify their email address and phone number and provide their name.
2. After creating an account, prospective participants are screened for eligibility via a web-based survey. Those meeting the inclusion criteria proceed to step 3.
3. Each participant then verifies their identity by providing a photo of themselves with a photo ID card or a link to a public social media profile that shows their name with a visible photo of themselves.
4. Participants who provide identity verification are invited to complete the baseline web-based survey.
5. Each participant is randomized after baseline completion, informed of their assignment, and provided with standardized web-based resources on PrEP access or brought to the first step of the PrEPTECH intervention.
6. All participants are invited to take the midpoint survey 90 days after the baseline survey.
7. All participants are invited to take the final survey 180 days after the baseline survey.

### Data Collection and Management

Study data come from surveys completed by participants at three time points, namely (1) before randomization (baseline); (2) 3 months (90 days) after enrollment; and (3) 6 months (180 days) after enrollment, as well as study implementation records that document participant follow-up activities and participant progress through the intervention. Additional study data come from the laboratory tests and medical intake forms collected before the provision of the first PrEP prescription and approximately 90 days later, completed by participants in the intervention arm.

All survey data are collected digitally and entered directly by participants via embedded surveys hosted by Qualtrics (Qualtrics International, Inc) into the PrEPTECH study website. For participants in the intervention arm, laboratory test and medical intake information is collected via myLAB Box (laboratory test) and an embedded survey hosted by Qualtrics (medical intake). Medication prescription information is transferred electronically, using DrChrono (EverCommerce), to Ridgeway Pharmacy.

Participants receive automated email and SMS text message reminders to complete all surveys, with additional customized follow-up via email and SMS text message from the study team. To maximize the response rate and study retention, participants are compensated with web-based gift cards for the completion of each survey: US $40 at baseline, US $50 at 3 months, and US $60 at 6 months.

Upon randomization, each participant was assigned a unique identifier used throughout the study. However, data will be associated with personal identifiers while the study is ongoing, as this is necessary for participant follow-up. Electronic data will be stored for 3 years after the conclusion of the study. All data relevant to the study will ultimately reside in ETR Associates’ Amazon Web Services system (Amazon Web Services, Inc) using Aptible (Aptible Inc) to ensure Health Insurance Portability and Accountability Act compliance.

### Outcomes

#### Primary Outcomes

PrEP initiation at 3 months after enrollment will be assessed using participant self-report of having taken at least one dose of PrEP medication between study enrollment and midpoint survey administration.

#### Secondary Outcomes

##### PrEP Initiation

PrEP initiation at 6 months after enrollment will be assessed using participant self-report of having taken at least one dose of PrEP medication at any point during the intervention period.

##### PrEP Persistence

PrEP persistence at 3 and 6 months after enrollment will be assessed using participant self-report of having taken at least one dose of PrEP medication during both the first and second 90-day periods of the intervention.

##### PrEP Adherence

PrEP adherence at 3 and 6 months after enrollment will be assessed through multiple measures. Continuous adherence will be assessed using participant self-report of the number of doses of PrEP taken in the past 30 days, excluding participants taking event-based PrEP, as adherence would need to be conceptualized differently for this approach to PrEP administration. Categorical adherence will be assessed using participant self-report of taking a dose of PrEP medication for at least four days per week, “all of the last 4 weeks (4 out of 4 weeks)” or “most of the last 4 weeks (3 out of 4 weeks),” excluding participants taking event-based PrEP.

##### PrEP Coverage

PrEP coverage at 3 and 6 months after enrollment will be assessed using participant self-report of the number of instances in the past 30 days of vaginal or anal sex in which there was no protection from event-driven PrEP (defined as taking PrEP between 2 and 24 hours ahead of the sex act and ≥2 doses 24 and 48 hours after the first dose), continuous PrEP (defined as taking a minimum of 4 doses of PrEP within a week of the sex act), or long-acting PrEP (defined as taking injectable PrEP within 2 months before the sex act).

##### PrEP Medication Prescription

PrEP medication prescription at 3 and 6 months after enrollment will be assessed through multiple measures, including self-report of having received a PrEP prescription at any point during the intervention, as well as documented medication prescription through study electronic health records (among eligible intervention arm participants only).

##### PrEPTECH Acceptability and Feasibility

The acceptability and feasibility of PrEPTECH as a telehealth HIV PrEP care intervention for young cisgender men and transgender women who have sex with men will be assessed through multiple measures, including the likelihood of recommending PrEPTECH; preference for continuing PrEPTECH beyond the study; and other variables assessing the perceived trustworthiness, privacy, helpfulness, and convenience of and comfort with PrEPTECH. We will also calculate a mean System Usability Scale score, which is a standard scale for evaluating web apps [45], and conduct a thematic analysis of responses to 4 free-text questions about participants’ perception of and experience with the PrEPTECH intervention and barriers to PrEP access encountered during the study. We will also examine clinical data, data on study attrition and completion, and other data from study implementation records.

##### Demographics

Participants’ age, sex, gender, gender identity, sex partners’ gender, sexual orientation, race, ethnicity, educational attainment, and living situation (living alone or with housemates or family) and whether languages other than English are spoken at participants’ home will be assessed.

##### Moderators and Mediators

The following parameters will be assessed at baseline as potential moderators of PrEP uptake: history of PrEP use, HIV risk perception (assessed using the Perceived Risk of HIV Scale) [46], PrEP attitudes (assessed using a PrEP attitude and stigma scale with scores ranging from 10 to 50) [47], behaviors associated with HIV risk (eg, instances of anal intercourse without a condom in the past 90 days), health insurance status, and reasons for enrolling in the study (using a predefined list of possible reasons formulated by the study staff with input from the CAB). A series of questions assess the barriers to PrEP access participants may have encountered during the study period as a potential mediator of differences in PrEP uptake. Participants are asked to attest to whether they have encountered specific PrEP access barriers compiled in consultation with the CAB and with reference to the literature falling into the categories of financial or insurance coverage, health care access, medical concerns, privacy, PrEP stigma, and practical considerations (including loss, theft or selling of medication). A single item on the 2 follow-up surveys will also ask all participants to rate whether they have a source of PrEP that meets their needs.

### Data Analysis

#### Overview

We will report the participant flow through the trial and the results in line with the CONSORT (Consolidated Standards of Reporting Trials) guidelines [48]. All analyses will be carried out following intention-to-treat principles. Data from participants will be allocated to the arm they were initially assigned to, regardless of whether they complete the intervention.

Data analysis will be conducted in SPSS (version 29 or higher; IBM Corp). Descriptive statistics will be calculated to describe the sample at baseline. Binary and categorical variables will be assessed through counts and percentages, and continuous variables will be assessed through means and SDs (medians will be computed for highly skewed variables). To assess baseline equivalence between the intervention and control arms of the study, regression analyses will be performed using age, race, ethnicity, gender, and educational attainment as dependent variables, with intervention condition as the independent variable. The groups will be considered not equivalent at baseline if a given variable differs significantly (*P*<.05) between the intervention arms. Differential and overall attrition will also be assessed using the What Works Clearinghouse recommended approach [49]. We expect ≤5% of the data to be missing; therefore, a complete case analysis is planned with no outcome or baseline imputation. However, if missing data go beyond 5%, then multiple imputation will be explored and run using the *mice package* in R (R Foundation for Statistical Computing) [50].

#### Primary and Secondary Outcome Analyses

##### Objective 1

To assess the effectiveness of PrEPTECH in enhancing PrEP uptake in comparison with the control condition, multiple logistic regression will be applied with the primary outcome (taking at least one dose of PrEP in the last 90 days, a dichotomous variable) as the dependent variable. The model will also include indicator variables denoting (1) the intervention group, (2) the number of days between the baseline and follow-up surveys (if there is a large range, which we do not expect), (3) the standard demographics of age at baseline and race and ethnicity at baseline, and (4) a set of a priori demographics and outcome-related covariates that screened positively as candidates for inclusion. A priori covariates that will be screened as candidates for inclusion into the model include gender, educational attainment, health insurance status, state, location, language spoken at home, sexual orientation, gender of sex partners, sexual risk factors, and the participants’ relationships with the persons they are living with. If there is high correlation (*r*>0.50) between some of these indicators, they will be excluded from the model. Bivariate analyses will be used to test for relationships between the outcome variable and potential covariates, with a threshold of *P*<.15. The standardized effect size of the intervention on PrEP uptake will also be calculated to determine the magnitude of the effect of the intervention.

##### Objective 2

To evaluate the acceptability of PrEPTECH as a telehealth HIV PrEP care intervention for young cisgender men and transgender women who have sex with men, we will assess participants’ likelihood of recommending PrEPTECH; participants’ preference for continuing PrEPTECH beyond the study; and other variables assessing the perceived trustworthiness, privacy, helpfulness, and convenience of and comfort with PrEPTECH among those in the intervention group. The percentage of participants expressing agreement or strong agreement with each item will be calculated. The System Usability Scale score will be calculated according to published guidelines to obtain a score out of 100 [45]. In addition, free-text answers to 3 questions about the benefits and drawbacks of PrEPTECH and how participants would characterize the intervention to a friend will be analyzed using a thematic approach.

The feasibility of PrEPTECH as a telehealth HIV PrEP care intervention for young cisgender men and transgender women who have sex with men will be assessed through a review of the study records. Clinical data will be used to assess loss to follow-up at various intermediate points in the intervention (eg, failure to return at-home laboratory kits or complete asynchronous medical intake forms). Records of clinical communications, written prescriptions, and efforts to assist intervention arm participants and to remind them to complete intervention steps will also be reviewed, with the number of contacts required at various stages calculated.

##### Objective 3

To examine the effects of PrEPTECH on adherence to PrEP, we will examine continuous measures. In each case, a multiple linear regression model will be applied. As described under *Objective 1*, the model will include (1) the intervention group, (2) the number of days between the baseline and follow-up surveys (if applicable), (3) the standard demographics of age at baseline and race and ethnicity at baseline, and (4) a priori demographics and outcome-related covariates that screened positively as candidates for inclusion. Variables will be screened in as described earlier.

The standardized effect size of the intervention on PrEP adherence will also be calculated to determine the magnitude of the effect of the intervention.

##### Objective 4

To examine the moderators of PrEP use, including HIV risk perception, PrEP attitudes, indications of sexual risk, insurance status, and reason for enrolling in the study, we will create multiple logistic regression models with PrEP uptake at 3 and 6 months as the outcome variable, intervention group as the predictor, and the potential moderator as an interaction term. The moderator will be considered statistically significant if *P*<.05.

A more detailed statistical analysis plan will be agreed upon before final analysis.

### Ethical Considerations

This study has been approved by the institutional review board (number 0000283) of ETR Associates and is registered on ClinicalTrials.gov (NCT04902820) [51].

## Results

This study was funded in 2019 with institutional review board approval in 2020. The PrEPTECH intervention was built over the next 1.5 years. Study recruitment was launched in February 2022 and completed in September 2022, with 229 participants recruited in total. Data collection was completed in April 2023, and analysis will be complete in September 2023. We expect to publish the results by the end of 2023.

## Discussion

### Overview

Despite the rapid expansion of telehealth services for PrEP in recent years, formal and rigorous evidence of their effectiveness is sparse. We describe the protocol for an RCT to assess the effectiveness, acceptability, and feasibility of a telehealth HIV PrEP care intervention, PrEPTECH, in increasing PrEP uptake among young cisgender men and transgender women who have sex with men. We hypothesize that participants in the intervention arm will show higher rates of PrEP uptake at both 3 and 6 months than those in the control arm and that PrEPTECH is a feasible and acceptable telehealth HIV PrEP care intervention for young cisgender men and transgender women who have sex with men.

### Limitations

One key limitation of this study is that it will rely on self-report data for PrEP uptake and adherence. In self-report, study participants may tend to overestimate their PrEP use. The use of electronic monitoring (such as medication event monitoring systems) and measurement of plasma or blood concentration yield more reliable measures of true PrEP use [52,53]; however, these were cost prohibitive for this study. Because we are using the same measurement approaches across both randomized groups, we do not anticipate differential bias across the study arms.

It is also worth noting that based on previous research from the PrEPTECH pilot study [36], in our original study proposal, we assumed 50% PrEP uptake in the control population and, therefore, calculated a sample size target of 400 participants, anticipating retaining 80% (n=320) of these participants at 180-day follow-up. However, 6 months into data collection, we revised our sample size calculations using the latest data on PrEP uptake in relevant populations and comparable research projects, including ongoing RCTs of digital interventions, to ensure that the participant sample size was adequate yet feasible given the resources required to enroll and retain participants.

### Conclusions

The study findings will be disseminated through open-access journals and academic conferences. A summary of the results will also be shared with all community partners, CAB members, and study participants and published via our agency’s website, newsletter, and social media channels. We anticipate that the study results will offer valuable evidence about the effectiveness, acceptability, and feasibility of telehealth HIV PrEP care interventions in this population.

## Appendix

Multimedia Appendix 1 Sample screenshots of the PrEPTECH intervention.

## Abbreviations

CAB: community advisory board
CONSORT: Consolidated Standards of Reporting Trials
LGBTQ+: lesbian, gay, bisexual, transgender, and queer
PrEP: pre-exposure prophylaxis
RCT: randomized controlled trial
STI: sexually transmitted infection

## Data Availability

The data sets generated and analyzed during this study are not publicly available because of participant consent restrictions. However, the data sets are available from the corresponding author upon reasonable request.
