# Supplementary material for: Effectiveness, Acceptability, and Feasibility of a Telehealth HIV Pre-Exposure Prophylaxis Care Intervention Among Young Cisgender Men and Transgender Women Who Have Sex With Men: Protocol for a Randomized Controlled Trial
Source: JMIR Res Protoc. 2023 Sep 15;12:e47932. doi: 10.2196/47932 (PMC10541640; doi:10.2196/47932)
Supplement: Multimedia Appendix 1 [file resprot_v12i1e47932_app1.pdf]

PrEPTECH

PrEPTECH is a paid study to see if an online program will help people start and continue taking PrEP.

Join the Study

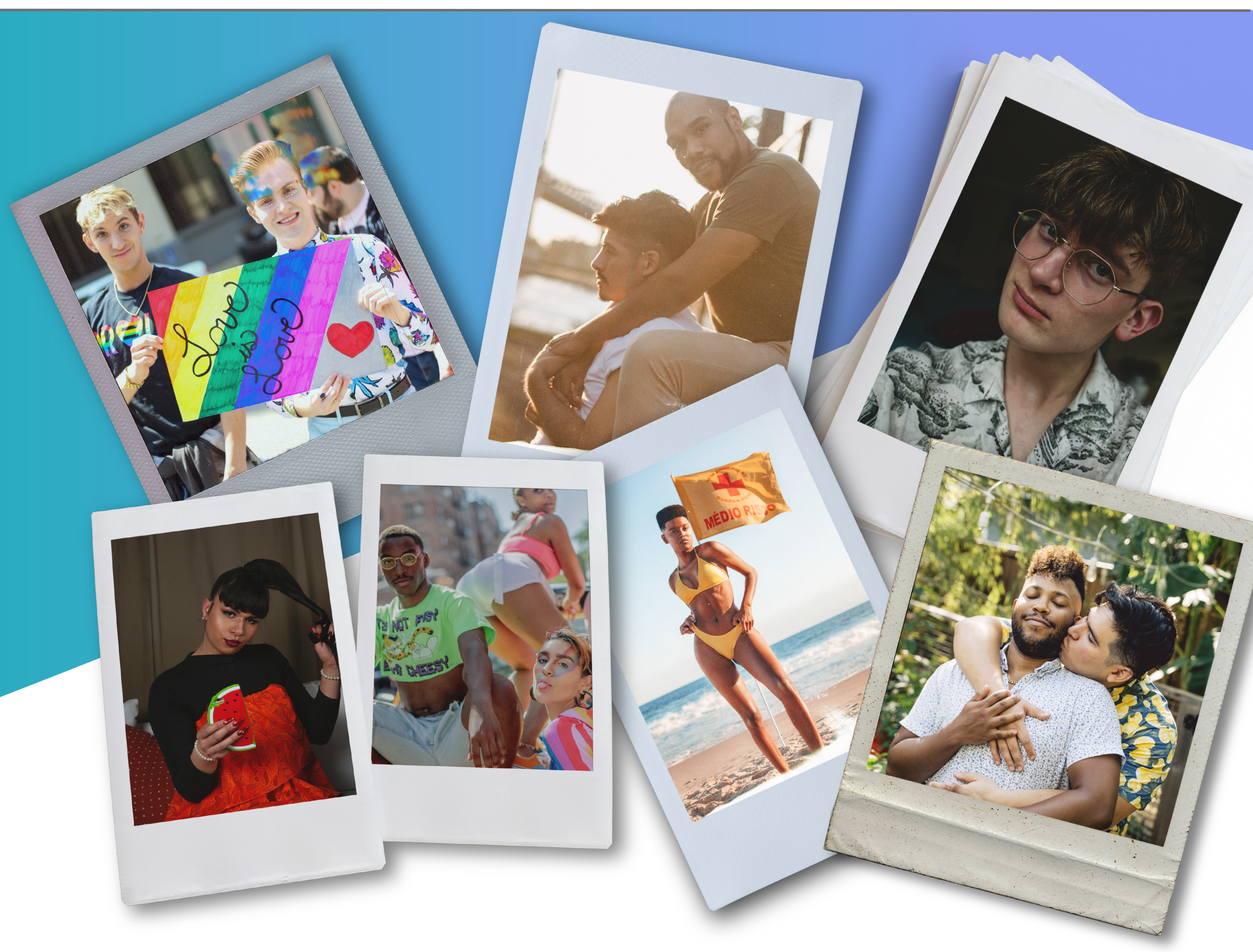

To get PrEP – a daily pill that prevents HIV – you usually need to see a doctor, go to a lab for tests, and pick up your meds at a pharmacy.
PrEPTECH changes all that!

What is the PrEPTECH Study?

We already know that PrEP works – this medication-based approach is a great way to prevent HIV— but we want to see if an online program makes it easier for people to start PrEP and remember to take their medication daily.

We are currently looking for young gay or bi cis men (cis means not trans) and trans women in California or Florida to join our PrEPTECH study. This is a randomized study, meaning participants will be split into two groups, purely based on chance. One group will receive access to PrEPTECH. The other won't.

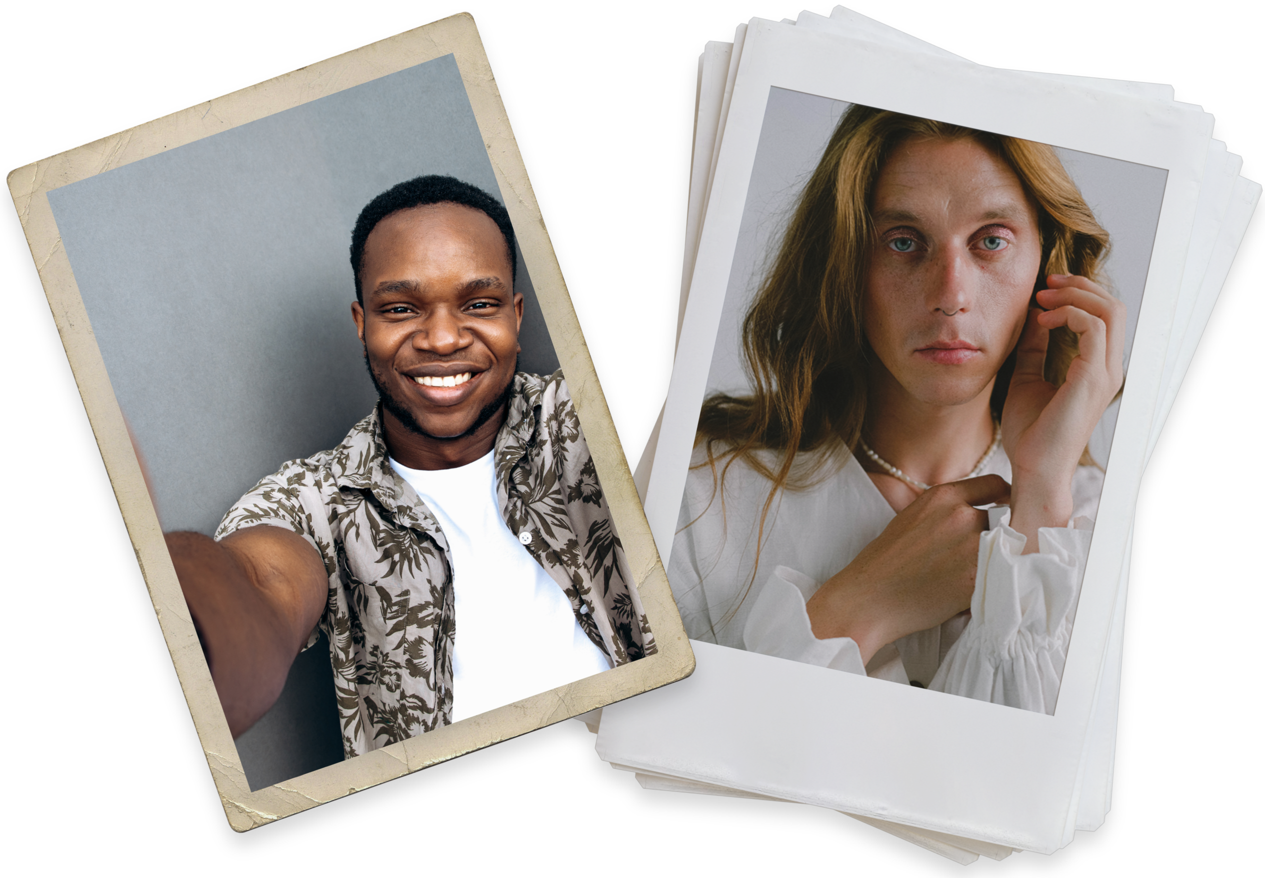

Everyone who participates in the study will be asked to complete 3 surveys and be compensated per survey – up to $150 total.

Join the Study

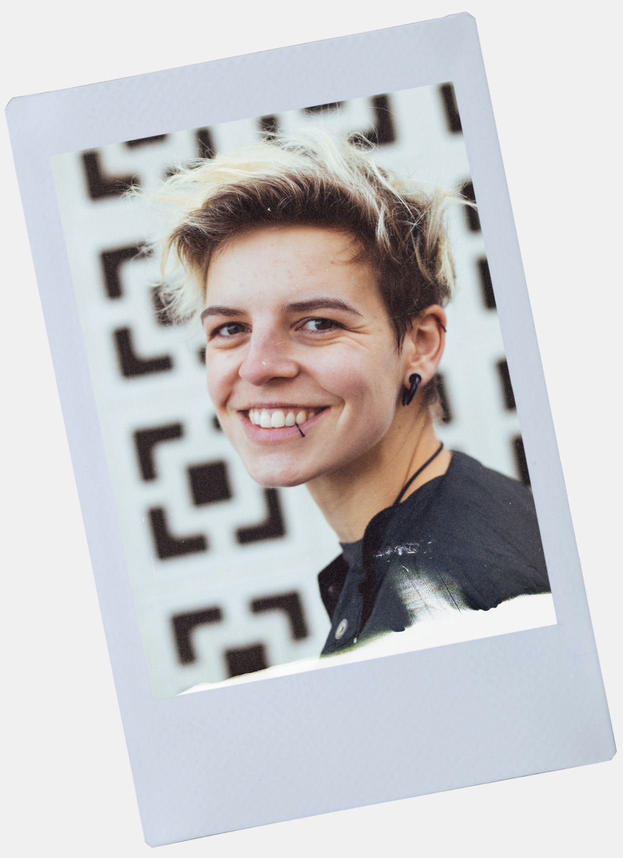

If you are assigned to the PrEPTECH group

You will receive:

+ Free at-home lab testing to check that PrEP is right for you.
+ Free consultation with our study doctor online or over the phone.
+ Free PrEP medication for 30 days sent directly to the address you choose. *If you are under 18 or a trans woman, you'll get free PrEP for the entire 6-month study period.*

Our study coordinator will help you figure out how to pay for PrEP once your free supply runs out. Our website will also help you manage your health while in the study by reminding you to take your PrEP.

If you aren't assigned to the PrEPTECH group

You will receive access to a list of online resources. So you will be to access PrEP on your own, but you won't receive direct help getting it.

If you're thinking about starting PrEP, but it seems complicated and time-consuming, then PrEPTECH may be right for you.

Study FAQs

Let's get social!

PrEPTECH has downloadable assets as well as suggested hashtags and captions, available for Instagram, Facebook, and Twitter.

We also have print materials available for you to share in your own community.

Social Outreach

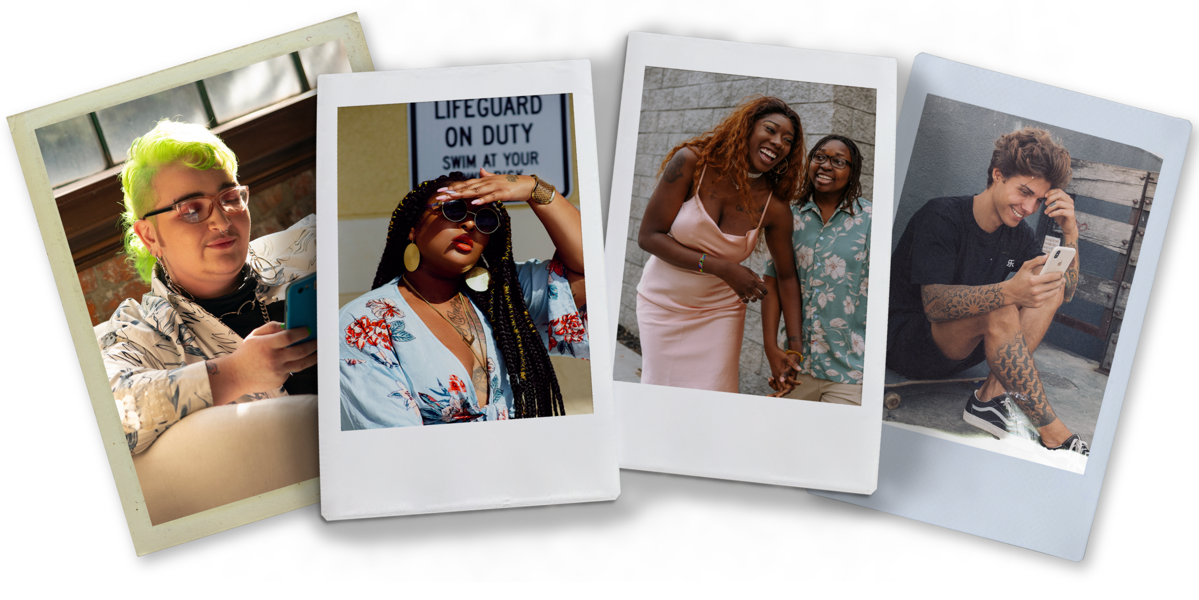

yth youth tech health
an etr. initiative

PrEPTECH is run by the YTH Initiative of ETR, a non-profit organization committed to improving health outcomes and advancing health equity for youth, families, and communities.

PrEPTECH

© 2020 YTH. All rights reserved.

Consent Information and Quiz

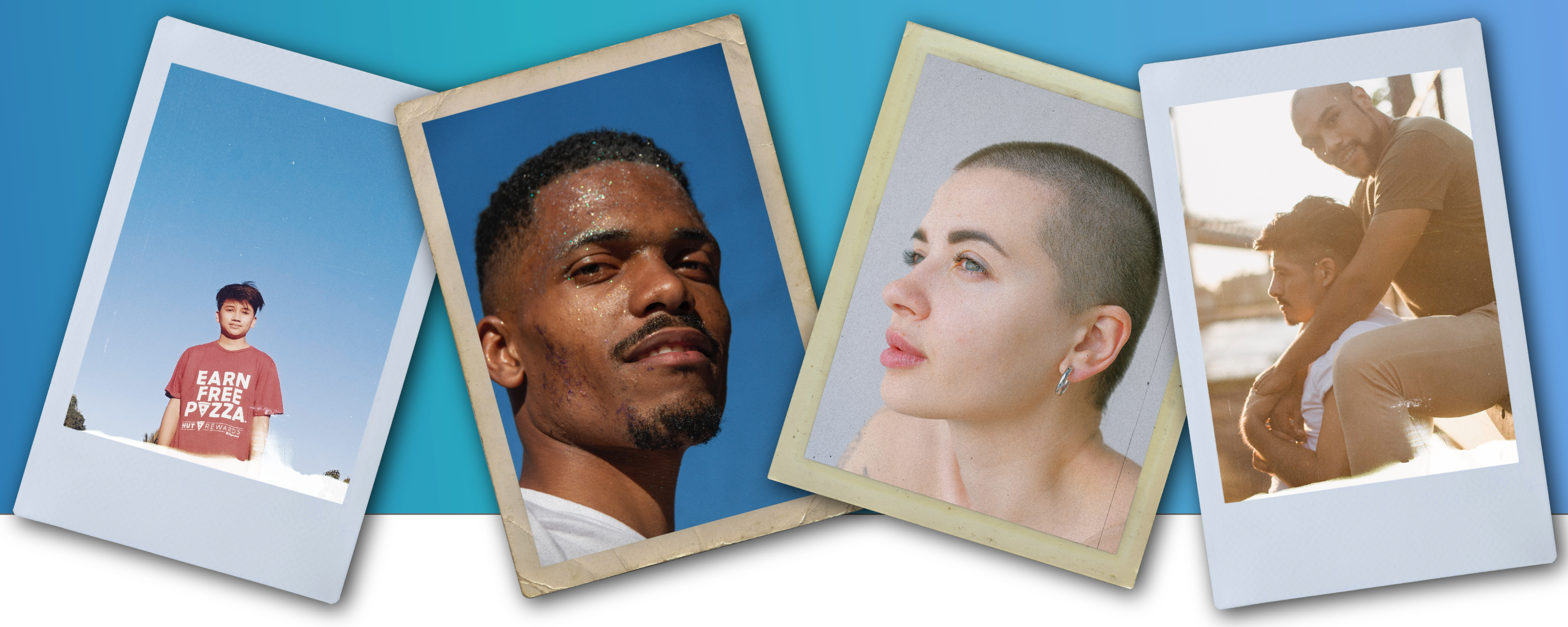

The Consent Process

Before you can participate in the study, we need your permission.

We are going to describe how the study works in detail, so you can decide whether or not you'd like to join. Along the way, there will be 5 quiz questions to make sure you understand the most important points. If you get a question wrong, review the information again then give it another try! When you get it right, you can move on to the next section.

What is this study about?

PrEP is medication approved by the U.S. Food and Drug Administration (FDA) to prevent HIV. It can decrease the chances that a person who is at risk of HIV will become HIV positive. In this study, we want to learn if an online program called PrEPTECH can help people start and keep taking PrEP, specifically a medication called Truvada.

1. What is this study about?

- Learning whether PrEP prevents HIV
- Learning whether PrEPTECH helps people start PrEP
- Learning whether PrEP causes side effects in young people
- Learning whether PrEP is more helpful for teens or adults

Question 1 of 5

Next

© 2020 YTH. All rights reserved.

PrEP Knowledge Quiz

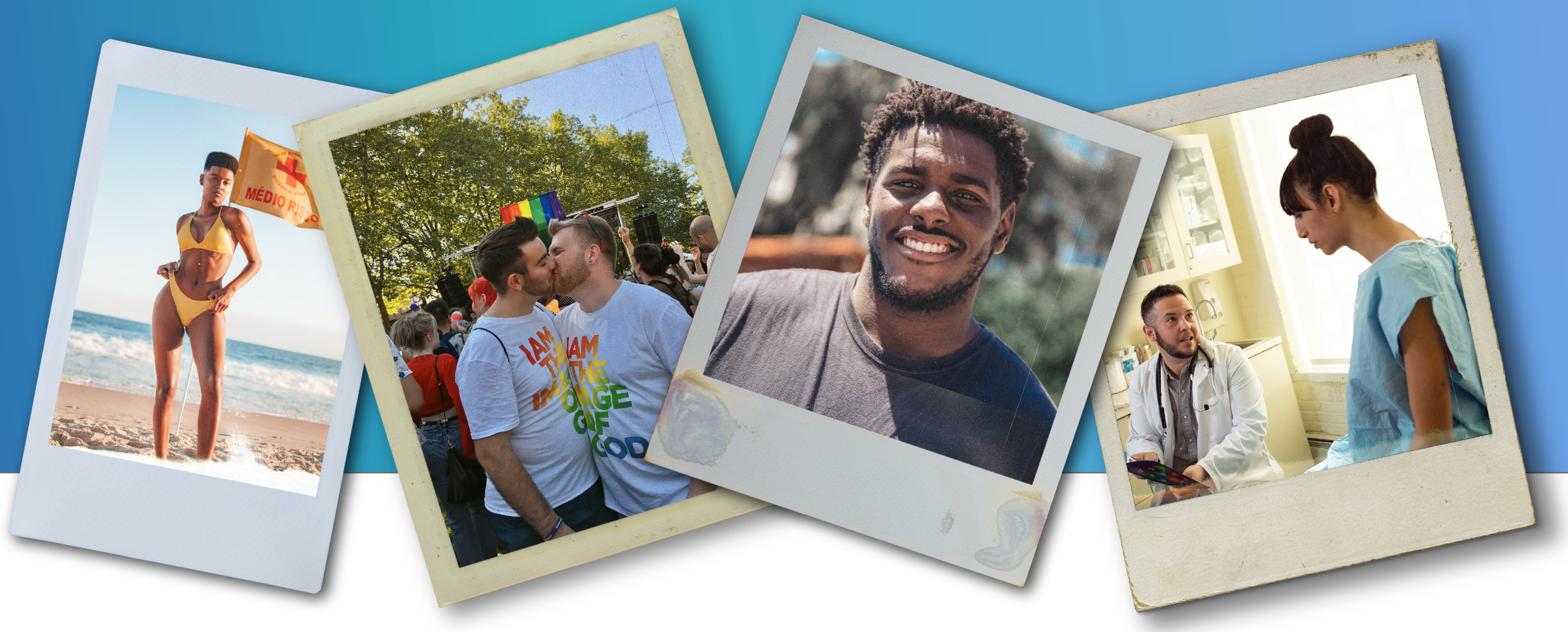

Does PrEP protect against other sexually transmitted infections (STIs) or pregnancy?

Nope. PrEP ONLY protects against HIV. We recommend using condoms to protect against STIs and to make sure birth control is part of the equation if you have sex with someone who could get pregnant and you and your partner want to avoid pregnancy.

3. True or False: PrEP protects you from other sexually transmitted infections (STIs) and pregnancy.

- True
- False

Question 3 of 8

Back | Next

© 2020 YTH. All rights reserved.

Welcome (Name)

Testing Time

Before we get you started on PrEP, we need to make sure it's right for you. You'll need tests for HIV, Hepatitis B, and STDs, as well as your liver and kidney functions, to make sure they're healthy. These tests do not mean that PrEP will harm your liver and kidneys; they are routine tests to make sure the drug is right for you. Don't worry though, these can all be done with simple blood and swab sample collections through a kit mailed to you!

Enter **123456789** to verify you are part of our study. Once you enter this code, the cost will drop to $0.

Order Your Kit

When your test kit arrives, we are here to help

Lab Kit Instructions and Tips

If you've already ordered your kit, it should be on its way. And if you have returned your kit, thanks so much! Typically, the lab kit takes 3-5 days to arrive once it's ordered and results from the lab take 1-2 weeks. If you've been waiting longer than that for the kit or your lab results, please feel free to reach out to the study team to see what's up. You can call or text **(510) 628-6434** or **email preptech@etr.org** to get help.

© 2020 YTH. All rights reserved.

Welcome (Name)

You will be taking 3 surveys over the course of the study, one at the start of the study, one after 90 days (or 3 months), and the one after 180 days (or 6 months). You will receive a gift card for completing each survey: $40 for the 1st survey, $50 for the 2nd survey, and $60 for the 3rd survey. Below is a record of which surveys you've taken, which surveys are upcoming, or are ready for you to take now. A reminder will be sent to you by text message or email bringing you to this page when it's time to take a survey.

Day 1

Baseline Survey

Completed Taken on 5/6/2020

Day 90

Mid-point Survey

Ready

Day 180

Final Survey

Not Time Yet

© 2020 YTH. All rights reserved.

Welcome (Name)

Use PrEPTECH to help you remember to take your PrEP daily. You can customize your reminder below.

Reminder Name* [Please enter your reminder name]

Time Zone* [India]

How often?* [One time per day]

Reminder time* [9] [00] [AM]

How do you want reminders?*

- [ ] Text
- [ ] Email

Personalize your reminder message:

PrEPTECH: Hey! It's time to take your pill :)

Submit

© 2020 YTH. All rights reserved.

Welcome (Name)

PrEP is pricey! This study will provide you with 30 days of free PrEP meds.

After that we will need to ID another payment option. Luckily, there are several ways to pay for PrEP and we can help you identify them. To get started, please tell us:

Are you currently covered by any health insurance or health coverage plan?

- ○ Yes
- ◉ No

You're uninsured.
OK, don't sweat it. Uninsured folks can definitely still get PrEP.

Option #1

Your first great option for paying for PrEP is called Ready, Set, PrEP, a government program that provides PrEP for free. As long as you don't have prescription drug insurance and get a negative HIV test and PrEP prescription through PrEPTECH, you can use this program to pay for your drugs and continue with PrEPTECH.

Ready, Set, PrEP requirements:

- You must have no insurance, test negative for HIV, and live in the US.
- You can use Ready, Set, PrEP no matter how high or low your income is.
- You don't need to be a citizen to enroll in Ready, Set, PrEP and participation doesn't count towards "public charge" designation, so it's safe no matter your immigration status.

You can enroll in Ready, Set, PrEP yourself or give the PrEPTECH permission to apply on your behalf.

Option #2

You can also get connected to a local community partner that can help you navigate care where you live. We have relationships with community groups in your area that would be glad to help you enroll in a program like Ready, Set, PrEP and get into care.

If you opt to do this, you won't receive home delivered PrEP through PrEPTECH and may need to go in for in-person labs or doctor's visits depending on the policies of the local PrEP navigators, but we'll still invite you to take the next two paid study surveys and keep you in study.

How would you like to get PrEP?

○ **Have the PrEPTECH team apply for Ready, Set, PrEP on my behalf**

- You'll give us permission to share your information and the last four digits of your Social Security number. We take care of the rest.

○ **Apply for Ready, Set, PrEP myself by phone or email.**

- You can fill out an online form or call a toll free number to apply.

○ **Ask for help getting free PrEP from a local patient navigator**

- We'll provide you a list of some excellent community organizations in your area that are ready to help.

Back | Submit

© 2020 YTH. All rights reserved.
